# Risk Factors for Recent HIV Infections among Adults in 14 Countries in Africa Identified by Population-Based HIV Impact Assessment Surveys, 2015–2019

**DOI:** 10.3201/eid2911.230703

**Published:** 2023-11

**Authors:** Dustin W. Currie, Christine A. West, Hetal K. Patel, Jennifer Favaloro, Fred Asiimwe, Felix Ndagije, Rachel Silver, Owen Mugurungi, Judith Shang, Clement B. Ndongmo, Daniel B. Williams, Edington Dzinotyiweyi, Anthony Waruru, Munyaradzi Pasipamire, Harriet Nuwagaba-Biribonwoha, Sindisiwe Dlamini, Natasha McLeod, Eugenie Kayirangwa, Gallican Rwibasira, Peter A. Minchella, Andrew F. Auld, Rose Nyirenda, Yimam Getaneh, Ashenafi Haile Hailemariam, Isabelle Tondoh-Koui, Natacha Kohemun, George S. Mgomella, Prosper Faustine Njau, Wilford L. Kirungi, Ibrahim Dalhatu, Kristen A. Stafford, Stephane M. Bodika, Faith Ussery, Stephen McCracken, Paul Stupp, Kristin Brown, Yen T. Duong, Bharat S. Parekh, Andrew C. Voetsch

**Affiliations:** Centers for Disease Control and Prevention, Atlanta, Georgia, USA (D.W. Currie, C.A. West, H.K. Patel, J. Favaloro, R. Silver, S.M. Bodika, F. Ussery, S. McCracken, P. Stupp, K. Brown, B.S. Parekh, A.C. Voetsch);; Centers for Disease Control and Prevention, Maseru, Lesotho (F. Asiimwe);; Columbia University, Maseru (F. Ndagije);; Zimbabwe Ministry of Health and Child Care, Harare, Zimbabwe (O. Mugurungi);; Centers for Disease Control and Prevention, Yaoundé, Cameroon (J. Shang, C.B. Ndongmo);; Centers for Disease Control and Prevention, Windhoek, Namibia (D.B. Williams);; Republic of Namibia Ministry of Health and Social Services, Windhoek (E. Dzinotyiweyi);; Centers for Disease Control and Prevention, Nairobi, Kenya (A. Waruru);; Centers for Disease Control and Prevention, Mbabane, Eswatini (M. Pasipamire);; Columbia University, Mbabane (H. Nuwagaba-Biribonwoha); Ministry of Health Mbabane, Mbabane (S. Dlamini);; Columbia University, New York, New York, USA (N. McLeod, Y.T. Duong);; Centers for Disease Control and Prevention, Kigali, Rwanda (E. Kayirangwa);; Republic of Rwanda Ministry of Health, Kigali (G. Rwibasira);; Centers for Disease Control and Prevention, Lusaka, Zambia (P.A. Minchella, A.F. Auld);; Ministry of Health, Lilongwe, Malawi (R. Nyrenda);; Ethiopian Public Health Institute, Addis Ababa, Ethiopia (Y. Getaneh);; Zhejiang University, Hangzhou, China (Y. Getaneh);; Centers for Disease Control and Prevention, Addis Ababa (A.H. Hailemariam);; Cote d’Ivoire Ministry of Health, Abidjan, Côte d’Ivoire (I. Tondoh-Koui);; Centers for Disease Control and Prevention, Abidjan (N. Kohemun);; Centers for Disease Control and Prevention, Dar es Salaam, Tanzania (G.S. Mgomella);; United Republic of Tanzania Ministry of Health, Dodoma, Tanzania (P.F. Njau);; Republic of Uganda Ministry of Health, Kampala, Uganda (W.L. Kirungi);; Centers for Disease Control and Prevention, Abuja, Nigeria (I. Dalhatu);; University of Maryland, Baltimore, Maryland, USA (K.A. Stafford)

**Keywords:** HIV, sexually transmitted diseases, cross-sectional studies, Population-Based HIV Impact Assessment, PHIA, HIV/AIDS and other retroviruses, sexually transmitted infections, viruses, zoonoses, Lesotho, Zimbabwe, Cameroon, Namibia, Kenya, Eswatini, Rwanda, Zambia, Malawi, Ethiopia, Côte d’Ivoire, Tanzania, Uganda, Nigeria

## Abstract

Identifying persons who have newly acquired HIV infections is critical for characterizing the HIV epidemic direction. We analyzed pooled data from nationally representative Population-Based HIV Impact Assessment surveys conducted across 14 countries in Africa for recent infection risk factors. We included adults 15–49 years of age who had sex during the previous year and used a recent infection testing algorithm to distinguish recent from long-term infections. We collected risk factor information via participant interviews and assessed correlates of recent infection using multinomial logistic regression, incorporating each surveyʼs complex sampling design. Compared with HIV-negative persons, persons with higher odds of recent HIV infection were women, were divorced/separated/widowed, had multiple recent sex partners, had a recent HIV-positive sex partner or one with unknown status, and lived in communities with higher HIV viremia prevalence. Prevention programs focusing on persons at higher risk for HIV and their sexual partners will contribute to reducing HIV incidence.

Sub-Saharan Africa has the highest HIV infection incidence and prevalence in the world (*1**,**2*). Although incidence is declining (*3*), more progress is needed to reduce transmission to a sufficient level that achieves global epidemic control. Several metrics for epidemic control have been proposed, including an incidence:mortality ratio (number of new HIV infections:total number of deaths from all causes among HIV-infected persons), a metric used providing that both new infections and deaths are low and declining (*4*). In sub-Saharan Africa, the Joint United Nations Programme on HIV/AIDS (UNAIDS) estimates that women and girls accounted for 63% of all new HIV infections in 2021 (*2*). Determining risk factors for HIV acquisition in countries with generalized HIV epidemics can help identify appropriate groups for tailored prevention programming and can support testing for those at highest risk of acquiring infection.

Methods used to examine risk factors for HIV often compare HIV-negative and HIV-positive persons (prevalence) (*5**–**7*) or use longitudinal cohort studies that comprise HIV-negative persons at baseline (*8**–**11*). Each of those methods has drawbacks. The prevalence approach does not distinguish recent from long-term infections, making it difficult to determine whether risk factors preceded the infection (*12*). In addition, risk factors for HIV might change over time, and programmatic efforts need to assess who is at the highest risk of acquiring new infections to prevent transmission. Longitudinal cohorts can establish timing of infection; however, they require long follow-up periods and large sample sizes and are subject to attrition bias that might not be equal across risk factors (*12*).

Assays that distinguish recent from long-term HIV-1 infections present an opportunity to estimate HIV incidence and assess risk factors in cross-sectional population-based household surveys (*12**,**13*). The limiting-antigen (LAg) avidity enzyme immunoassay (EIA) has been well characterized, validated, and used for the detection of recent infections and estimation of HIV-1 incidence as part of a recent infection testing algorithm (RITA) in cross-sectional surveys, including Population-Based HIV Impact Assessment (PHIA) surveys (*14**,**15*). Therefore, PHIA surveys can identify risk factors for new HIV infections in the general population across multiple countries in sub-Saharan Africa by using the largest sample of recent infections. We identified demographic and behavioral risk factors for recent HIV infections among sexually active adults across 14 sub-Saharan Africa countries and assessed whether those factors differed between recent and long-term infections.

## Methods

### Study Design

PHIAs are nationally representative, cross-sectional, population-based surveys of households across each country (*16**,**17*). A 2-stage, stratified cluster sample design was used in each survey: enumeration areas were selected within strata (subnational units, such as regions) by using a probability proportional to size method, and households within enumeration areas were randomly selected in the second stage. Weights were calculated to account for unequal probability of household selection, nonresponse, and noncoverage. Within selected households, the household head completed a household survey, and eligible household members completed individual interviews and had blood collected after providing consent for each survey component.

### Study Population

We used data from PHIA surveys completed in 14 countries during 2015–2019: Cameroon, Cote d’Ivoire, Eswatini, Ethiopia, Kenya, Lesotho, Malawi, Namibia, Nigeria, Rwanda, Tanzania, Uganda, Zambia, and Zimbabwe. We pooled data across PHIAs in a multicountry analysis because of small sample sizes for recent infections within each country. We included adults 15–49 years of age who reported engaging in sexual activity during the year before their interview. We only included persons who consented to a blood draw and had a valid final RITA classification. 

### Variable Definitions

We explored demographic and behavioral variables collected during participant interviews to identify potential risk factors. Demographic factors were country, sex, age, marital status, education, and household wealth. Behavioral factors were number of recent sexual partners (during the previous 12 months), age of sexual debut, HIV status of partner(s), age of partner(s), condom usage, and voluntary medical male circumcision status. Age of sexual debut was divided into <18 or >18 years categories; 18 years of age was the median. The numbers of partners in the previous year were grouped into categories (0, 1, or >2 partners), consistent with previous literature and an examination of the data (*12**,**13*). Age groups were 15–24, 25–34, and 35–49 years, according to published precedent.

We calculated community viremia levels within each stratum in each country. We defined participants with long-term HIV infections and detectable viral load of >1,000 copies/mL as viremic and all HIV-negative or HIV-positive participants with an undetectable viral load as nonviremic. We excluded persons with recent HIV infection (as defined in the next paragraph) from community viremia calculations, which were calculated as the weighted number of viremic persons divided by the weighted number of viremic plus nonviremic persons within each stratum. We then categorized each stratum into quantiles representing the percentages of persons with nonsuppressed HIV infection within the stratum.

The 3 primary outcome categories for each participant were recent HIV infection, long-term HIV infection, or HIV negative. All participants were tested for HIV in the household according to each country’s national testing algorithm. Confirmatory HIV testing was completed in all countries except Uganda by using the Geenius HIV-1/2 rapid test (Bio-Rad Laboratories, https://www.bio-rad.com). We classified HIV according to confirmatory testing and excluded a small number of participants (n<25) who tested positive for HIV-2 but not HIV-1 from recency testing because the LAg-Avidity EIA is meant for HIV-1 recency classification only. Among HIV-positive participants, RITA was used to distinguish recent from long-term infections. The first step of RITA used the LAg-Avidity EIA (Sedia Biosciences Corp., https://www.sediabio.com, for plasma specimens or Maxim Biomedical, https://www.maximbio.com, for dried blood spot specimens), which assesses development of antibody avidity. We classified participants with a median normalized optical density <1.5 for plasma samples (or <1.0 for dried blood spot samples where venous blood could not be collected [<5% of participants]) as LAg-recent infections. Next, we categorized participants as recently infected if they had LAg-recent infections, HIV viral loads >1,000 copies/mL, and an absence of antiretroviral drug metabolites in their blood by using RITA (*14**,**15*). All HIV-1–positive participants who did not meet the criteria for recent infection were categorized as having long-term HIV infection.

### Statistical Analysis

Initial data analysis strategies were bivariate comparisons between HIV-negative participants and those who had a recent HIV infection or long-term HIV infections. We calculated an overall χ^2^ test statistic and unadjusted odds ratios for categorical variables. We used Taylor series weights and variables representing strata and units for all analyses. We performed analyses by using SAS version 9.4 (SAS Institute, https://www.sas.com) and considered p values <0.05 statistically significant.

Our model-building strategy incorporated candidate exposure variables with bivariate p values <0.20. In cases where variables were colinear, we included only 1 variable or set of variables in the final model. Only variables collected consistently across countries were eligible for the multivariable model. We used multinomial logistic regression to calculate adjusted weighted odds ratios accounting for the complex survey sample design.

### Ethics Statement

PHIA surveys were funded by the US President’s Emergency Plan for AIDS Relief (PEPFAR) (*18*); technical assistance was provided by the US Centers for Disease Control and Prevention. The surveys were conducted through cooperative agreements with grantees/federal entities, including country Ministries of Health, ICAP at Columbia University (New York, New York, USA), and the University of Maryland (Baltimore, MD, USA). Each survey was approved by human subject institutional review boards specific for each country, cooperative agreement grantees/federal entities conducting the survey, or the US Centers for Disease Control and Prevention.

## Results

### Population Description

Across the 14 countries included in this analysis, we identified 16,831 HIV-positive and 241,909 HIV-negative PHIA participants (Figure). Of the 16,831 HIV-positive participants, 264 (1.6% of all HIV-positive participants) had recent infections and 16,567 had long-term infections. Sample sizes of participants who met inclusion criteria ranged from 5,874 in Eswatini to 95,463 in Nigeria (Table 1). Biomarker (blood draw) response rates ranged from 86.7% in Malawi to 99.0% in Uganda for female participants and 85.3% in Namibia to 98.5% in Uganda for male participants.

**Figure:**
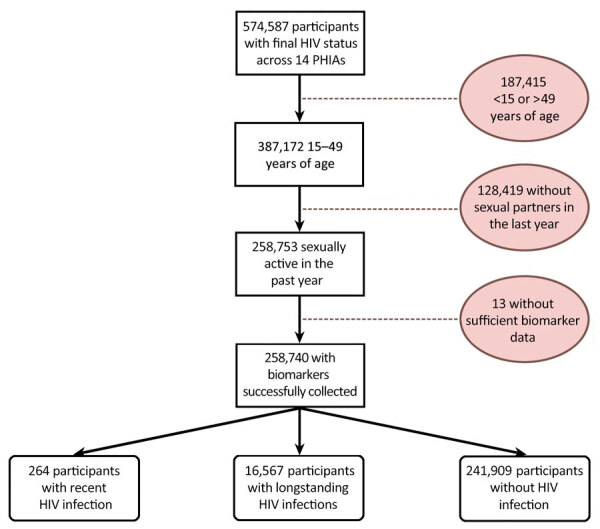
Inclusion and exclusion criteria and final outcome status in study of population-based assessments of risk factors for recent HIV infections among sexually active adults in 14 sub-Saharan Africa countries, 2015–2019. Shaded circles indicate numbers of excluded participants and reasons for exclusion from the study. Final outcome was categorized into 3 groups of participants: recent HIV infection, long-term HIV infection, and HIV negative. PHIA, Population-Based HIV Impact Assessment.

**Table 1:** Sample sizes of sexually active adults 15–49 years of age in study of risk factors for recent HIV infections among adults in 14 countries in Africa identified by Population-based HIV Impact Assessment surveys, 2015–2019*

Region and country	HIV negative	Long-term HIV infection	Recent HIV infection	Total sample size
Western Africa				
Cameroon	15,747	617	18	16,382
Cote d’Ivoire	11,688	258	4	11,950
Nigeria	93,895	1,537	31	95,463
Subtotal	121,330	2,412	53	123,795
Eastern Africa				
Ethiopia	8,131	265	4	8,400
Kenya	15,084	880	7	15,971
Tanzania	17,113	1,070	27	18,210
Rwanda	15,665	520	4	16,189
Uganda	17,850	1,171	30	19,051
Subtotal	73,843	3,906	72	77,821
Southeastern Africa				
Malawi	9,735	1,428	19	11,182
Zambia	9,762	1,486	30	11,278
Zimbabwe	9,663	1,976	21	11,660
Subtotal	29,160	4,890	70	34,120
Southern Africa				
Eswatini	3,848	2,004	22	5,874
Lesotho	5,480	2,107	31	7,618
Namibia	8,248	1,248	16	9,512
Subtotal	17,576	5,359	69	23,004
Overall total	241,909	16,567	264	258,740

*Sexually active adults are those who reported >1 sexual partner in the previous 12 mo.

### Bivariate Analysis Findings

We performed weighted bivariate comparisons between HIV-negative and recently infected participants (Table 2). Region was significantly associated with recent HIV infection; persons living in western Africa represented only 27.1% of persons with recent HIV infections but represented 51.5% of those who were HIV negative. In addition, female sex, age 25–34 years, and divorced or separated marital status were associated with recent HIV infection. Among young adults 15–24 years of age, female participants accounted for 72.1% of recent infections. Demographic variables not associated with recent infection were urban and rural locations, household wealth, and working during the previous 12 months.

**Table 2:** Bivariate comparisons of risk factors for HIV infections among adults in 14 countries in Africa identified by Population-Based HIV Impact Assessment surveys, 2015–2019

Risk factor	Recent HIV infection				HIV negative					Long-term HIV infections			
	No.	Weighted			No.	Weighted		p value*		No.	Weighted		p value†
		No.	%			No.	%				No.	%	
Region‡								<0.0001					0.01
Western Africa	53	31,539	27.1		121,330	72,879,939	51.5			2,412	1,348,293	21.1	
Eastern Africa	72	53,892	46.3		73,843	53,868,359	38.1			3,906	2,756,375	43.1	
Southeastern Africa	70	24,950	21.4		29,160	13,091,358	9.3			4,890	1,856,636	29	
Southern Africa	69	6,069	5.2		17,576	1,615,316	1.1			5,359	436,423	6.8	
Sex								0.005					0.92
M	78	40,782	35		99,363	66,646,015	47.1			5,000	2,261,024	35.3	
F	186	75,668	65		142,546	74,808,958	52.9			11,567	4,136,702	64.7	
Age group, y								0.03					<0.0001
15–24	91	36,543	31.4		67,966	41,540,180	29.4			1,893	755,870	11.8	
25–34	111	53,441	45.9		91,808	53,077,420	37.5			6,178	2,371,780	37.1	
35–49	62	26,466	22.7		82,135	46,837,373	33.1			8,496	3,270,077	51.1	
Household wealth								0.41					0.70
Lowest 40%	96	40,848	35.1		97,567	54,229,216	38.3			6,224	2,161,490	33.8	
Upper 60%	168	75,602	64.9		144,255	87,186,311	61.7			10,326	4,231,165	66.2	
Household location§													0.34
Urban	126	46,670	40.1		96,798	63,325,186	44.8	0.25		6,924	2,784,412	43.5	
Rural	138	69,780	59.9		145,111	78,129,786	55.2			9,643	3,613,314	56.5	
Education level								0.04					0.07
None	25	11,533	10.1		44,294	25,840,908	18.5			1,413	696,206	10.9	
Primary	100	49,596	43.6		86,089	52,676,177	37.7			7,676	3,299,037	51.8	
Postprimary	138	52,660	46.3		109,771	61,061,737	43.7			7,431	2,370,552	37.3	
Worked in previous 12 mo								0.94					0.58
Yes	127	62,728	53.9		123,378	76,651,794	54.2			7,911	3,318,448	51.9	
No	137	53,722	46.1		118,448	64,758,011	45.8			8,651	3,076,646	48.1	
Marital status								<0.0001					<0.0001
Married/like married	151	69,813	60.2		175,295	101,169,547	71.6			10,908	4,436,093	69.5	
Divorced/separated	41	22,659	19.5		10,826	6,561,523	4.6			1,952	836,072	13.1	
Widowed	3	388	0.3		2,054	1,092,655	0.8			968	362,925	5.7	
Never married	68	23,204	20		53,335	32,405,793	22.9			2,700	751,311	11.8	
Age of sexual debut, y								0.001					0.006
<18	157	66,675	59.9		106,632	63,470,656	46.3			7,653	3,134,550	50.2	
≥18	98	44,632	40.1		127,738	73,588,418	53.7			8,493	3,111,333	49.8	
No. sexual partners in previous 12 mo								0.0003					<0.0001
1 partner	200	82,904	71.2		204,635	116,558,470	82.4			13,953	5,238,471	81.9	
>2 partners	64	33,546	28.8		37,274	24,896,503	17.6			2,614	1,159,256	18.1	
Relationship with last sexual partner								0.05					0.005
Husband/wife or live-in partner	121	47,756	57.4		97,489	54,972,899	59.1			9,562	3,636,435	69.3	
Partner, not living with	56	16,609	20.0		19,979	9,938,239	12.5			2,924	742,195	14.1	
Ex-spouse or ex-partner	10	4,193	5.0		5,036	2,610,448	3.3			640	221,601	4.2	
Friend or acquaintance	25	13,507	16.2		15,229	10,823,253	13.6			1,117	556,196	10.6	
Other relationship	4	1,168	1.4		1,936	1,186,842	1.5			206	89,099	1.7	
Sexual partner outside marriage/live-in partner, previous 12 mo								<0.0001					<0.0001
All partners spouse/live-in	123	52,369	45.0		167,344	95,343,903	67.4			10,146	3,999,570	62.6	
Not all partners spouse/live-in	140	64,057	55.0		74,407	46,022,631	32.6			6,412	2,393,107	37.4	
Condom use at last sexual intercourse								0.18					0.0004
Used condom	71	21,302	19.0		37,519	20,655,616	15.1			6,554	1,940,679	31.5	
Did not use condom	186	90,769	81.0		197,907	116,224,955	84.9			9,595	4,223,093	68.5	
Condom use at last sexual intercourse with a non–spouse/live-in partner¶								0.02					0.001
Used condom	44	14,409	24.1		27,045	15,467,358	36.8			2,918	834,620	38.4	
Did not use condom	89	45,270	75.9		41,936	26,610,675	63.2			3,121	1,337,601	61.6	
HIV status of sexual partners in previous 12 mo								<0.0001					<0.0001
>1 partner thought/told/tested HIV+	17	9,272	8.0		2,460	1,223,899	0.9			5,415	2,050,692	32.4	
>1 partner with unknown HIV status	160	73,747	63.4		125,184	74,688,294	52.8			6,180	2,642,358	41.8	
All partners thought/told/tested HIV–	84	33,217	28.6		113,852	65,396,552	46.3			3,984	1,628,233	25.8	
Age difference with sexual partners								0.08					0.37
All partners <5 years older than participant	145	68,659	59.6		148,663	91,313,569	65.3			9,487	3,765,009	60.2	
>1 partner 5–9 years older than participant	68	26,805	23.3		44,307	23,554,393	16.8			3,476	1,254,666	20.1	
>1 partner >10 years older than participant	44	19,708	17.1		45,609	24,970,655	17.9			3,197	1,235,268	19.7	
Community HIV viremia#								<0.0001					0.03
Lowest quartile	18	14,601	12.5		63,635	45,382,548	32.1			1,022	533,366	8.3	
Second quartile	29	13,682	11.7		61,878	31,538,699	22.3			1,297	588,249	9.2	
Third quartile	65	36,361	31.2		63,050	38,179,235	27.0			3,452	1,786,811	27.9	
Highest quartile	152	51,806	44.5		53,346	26,354,490	18.6			10,796	3,489,301	54.5	

*p value comparing the distribution of the variable of interest among participants with recent HIV infection and those who were HIV-negative. †p value comparing the distribution of the variable of interest among participants with recent HIV infection and those with long-term HIV infections. ‡Western: Cameroon, Cote d’Ivoire, Nigeria; Eastern: Ethiopia, Kenya, Rwanda, Tanzania, Uganda; Southeastern: Malawi, Zambia, Zimbabwe; Southern: Eswatini, Lesotho, Namibia. §In Ethiopia, only the urban population was sampled. ¶Only calculated among participants with >1 sexual partner who was not a spouse or live-in partner. #Lowest quartile (0–25%), <39.38 viremic persons/10,000 population; second quartile (25%–50%), 39.38–122.51 viremic persons/10,000 population; third quartile (50%–75%), 122.52–297.03 viremic persons/10,000 population; highest quartile (76%–100%), >297.03 viremic persons/10,000 population.

Behavioral factors were also associated with the prevalence of recent infections. For example, compared with HIV-negative participants, those who had recent HIV infections were more likely to have had a sexual debut at <18 years of age, had >1 partner in the previous 12 months, had sex with a partner with whom they were not living, not used condoms during their last sexual intercourse with a nonregular partner, and had sex within the previous 12 months with a partner who had unknown HIV status or an HIV-positive status (Table 2).

We also performed weighted bivariate comparisons of recently infected participants and those with long-term infections (Table 2). Compared with participants 35–49 years of age, adolescents and young adults (15–34 years of age) had a greater percentage of recent infections than long-term infections. Participants who were never married or were divorced/separated had a greater percentage of recent than long-term infections, whereas those who were widowed were more likely to have long-term infections. Sex, employment, education, household wealth, and urbanicity were not associated with recent versus long-term infections. Behavioral factors more common in participants who had recent HIV infections (compared with those with long-term HIV infections) were sexual debut at <18 years of age, >1 sexual partner in the previous 12 months, having partners other than a spouse or live-in partner in the previous 12 months, not using a condom during their last sexual intercourse, and having a partner in the previous 12 months who had unknown HIV status (Table 2).

We performed bivariate exploratory comparisons of variables that were not collected consistently across all PHIAs or were only relevant to population subgroups (Table 3). Participants who had a sexually transmitted disease diagnosis or sexually transmitted infection symptoms and male participants who did not have a medical circumcision were more likely to be recently infected than HIV-negative. In addition, participants who had long-term infections were more likely to have previously been tested for HIV >1 year ago and were more likely to be uncircumcised than those who had a recent HIV infection (Table 3).

**Table 3:** Description of potential risk factors for HIV infection from data collected inconsistently across Population-Based HIV Impact Assessment surveys among adults in 14 countries in Africa, 2015–2019*

Variable	Recent HIV Infections				HIV negative					Long-term HIV infections			
	No.	Weighted			No.	Weighted		p value		No.	Weighted		p value
		No.	%			No.	%				No.	%	
Previous HIV testing†													
Never	29	12,504	13.3		28,497	18,070,566	21.2	0.08		950	12,504	8.7	0.0007
In previous year	120	40,316	42.8		63,272	34,907,993	41.0			4,941	1,852,308	33.3	
>12 mo ago	79	41,334	43.9		54,926	32,071,315	37.7			9,053	3,230,533	58.1	
Hazardous drinking‡													
Yes	23	6,465	12.3		7,687	3,782,225	10.7	0.61		1,286	464,592	14.8	0.38
No	115	46,171	87.7		53,395	31,645,051	89.3			8,038	2,671,216	85.2	
STD diagnosis§													
Yes	7	2,891	10.7		1,239	780,205	4.0	0.02		350	153,294	7.3	0.23
No	68	24,249	89.3		37,007	18,917,995	96.0			4,889	1,939,752	92.6	
STI symptoms¶													
Yes	24	14,602	27.0		9,189	7,347,057	14.3	0.007		1,448	790,794	21.6	0.28
No	81	39,535	73.0		59,901	46,925,388	85.7			5,602	2,865,735	78.4	
Victim of sexual violence by partner in previous 12 mo#													
Yes	2	869	3.8		424	217,821	1.5	0.17		60	20,445	1.4	0.15
No	77	21,816	96.2		33,334	14,227,044	98.5			5,650	1,422,172	98.6	
VMMC status**													
Medical	13	8,262	21.1		31,546	23,295,330	38.2	0.005		976	562,564	26.2	0.004
Traditional	26	16,358	41.7		36,456	26,060,244	42.7			1,028	508,445	23.7	
None	36	14,610	37.2		24,119	11,612,109	19.0			2,797	1,073,815	50.1	

*STD, sexually transmitted disease; STI, sexually transmitted infection; VMMC, voluntary medical male circumcision. †Excludes Nigeria. ‡Includes Eswatini, Kenya, Malawi, Namibia, Tanzania, Zambia, and Zimbabwe. §Includes Ethiopia, Kenya, Malawi, Zambia, and Zimbabwe. ¶Includes Ethiopia, Kenya, Malawi, Tanzania, Zambia, and Zimbabwe. #Includes a single participant per household in Cameroon, Cote D’Ivoire, Ethiopia, Lesotho, Malawi, Namibia, Eswatini, Uganda, Zambia, and Zimbabwe. **Men only.

### Multivariate Analysis Findings

In the adjusted model, the southern, southeastern, and eastern Africa regions and community-level viremia remained significantly associated with recent infections (Table 4). Participants living in countries within eastern, southeastern, and southern Africa had higher odds of recent HIV infection compared with those in western Africa, even after adjusting for community-level viremia. In addition, participants grouped in the third and fourth highest quartiles of community-level viremia were more likely to have a recent HIV infection than those in the lowest quartile; the odds of recent infection increased with each viremia quartile.

**Table 4:** Correlates of recent and long-term HIV infections compared with HIV negativity in study of risk factors for HIV infections among adults in 14 countries in Africa identified by Population-Based HIV Impact Assessment surveys, 2015–2019

Category	Recent infection vs. HIV-negative			Long-term infection vs. HIV-negative	
	Crude odds ratio (95% CI)	Adjusted odds ratio (95% CI)		Crude odds ratio (95% CI)	Adjusted odds ratio (95% CI)
Region					
Eastern Africa	2.26 (1.46–3.50)	1.88 (1.18–3.02)		2.74 (2.53–3.00)	1.73 (1.58–1.89)
Southeastern Africa	4.39 (2.86–6.73)	2.74 (1.64–4.57)		7.74 (7.17–8.35)	2.66 (2.42–2.93)
Southern Africa	8.58 (5.54–13.28)	4.73 (2.65–8.44)		15.02 (13.89–16.25)	4.03 (3.63–4.47)
Western Africa	Referent	Referent		Referent	Referent
Sex					
F	1.56 (1.09–2.24)	1.82 (1.11–2.98)		1.62 (1.55–1.70)	1.81 (1.68–1.96)
M	Referent	Referent		Referent	Referent
Age group, y					
15-24	1.60 (1.02–2.51)	1.26 (0.74–2.15)		0.27 (0.25–0.29)	0.26 (0.24–0.29)
25-34	1.68 (1.09–2.58)	1.43 (0.92–2.23)		0.64 (0.61–0.68)	0.64 (0.60–0.68)
35-49	Referent	Referent		Referent	Referent
Marital status					
Married/cohabiting	0.87 (0.58–1.31)	1.16 (0.67–1.99)		1.92 (1.79–2.07)	1.35 (1.21–1.51)
Divorced/separated/widowed	4.24 (2.48–7.26)	3.58 (1.92–6.69)		6.97 (6.37–7.62)	3.28 (2.91–3.70)
Never married	Referent	Referent		Referent	Referent
Age of sexual debut, y					
<18	1.74 (1.23–2.46)	1.42 (0.99–2.04)		1.17 (1.11–1.23)	1.20 (1.13–1.28)
≥18	Referent	Referent		Referent	Referent
No. sexual partners in previous 12 mo					
1 partner	Referent	Referent		Referent	Referent
>2 partners	1.95 (1.34–2.83)	1.92 (1.23–3.00)		0.99 (0.93–1.06)	1.05 (0.96–1.15)
Condom used at last sex					
Condom used	1.32 (0.88–1.99)	0.97 (0.58–1.62)		2.59 (2.45–2.73)	2.13 (1.97–2.30)
Condom not used	Referent	Referent		Referent	Referent
HIV status of sexual partners in previous 12 mo					
>1 partner thought/told/tested HIV+	15.15 (6.81–33.69)	7.25 (3.41–15.40)		68.92 (6.80–33.68)	42.74 (38.53–47.42)
>1 partner with unknown HIV status	1.90 (1.34–2.69)	2.05 (1.38–3.03)		1.41 (1.32–1.49)	1.73 (1.62–1.85)
All partners thought/told/tested HIV–	Referent	Referent		Referent	Referent
Age difference with sexual partners					
All partners <5 years older than participant	Referent	Referent		Referent	Referent
>1 partner 5–9 years older than participant	1.38 (0.95–2.02)	1.05 (0.67–1.66)		1.27 (1.20–1.35)	0.97 (0.90–1.06)
>1 partner >10 years older than participant	0.98 (0.65–1.48)	0.97 (0.58–1.62)		1.20 (1.13–1.27)	1.15 (1.06–1.25)
Community-level viremia*					
Lowest quartile	Referent	Referent		Referent	Referent
Second quartile	1.42 (0.64–3.13)	1.86 (0.79–4.38)		1.57 (1.38–1.80)	1.85 (1.62–2.11)
Third quartile	2.87 (1.36–6.08)	3.16 (1.38–7.26)		3.99 (3.56–4.48)	3.68 (3.27–4.13)
Highest quartile	6.58 (3.22–13.46)	4.81 (2.10–11.00)		11.38 (10.21–12.68)	6.84 (6.09–7.69)

*Lowest quartile (0–25%), <39.38 viremic persons/10,000 population; second quartile (25%–50%), 39.38–122.51 viremic persons/10,000 population; third quartile (50%–75%), 122.52–297.03 viremic persons/10,000 population; highest quartile (76%–100%), >297.03 viremic persons/10,000 population.

Individual demographic characteristics, including sex and marital status, remained significantly associated with HIV acquisition; female participants had 1.82 times greater odds of recent HIV infection than for male participants. In addition, participants who were divorced, separated, or widowed had 3.58 times greater odds of recent HIV infection than for those who were never married. Age group was not significantly associated with HIV acquisition risk in adjusted models (Table 4). Sexual behavior characteristics that remained significantly associated with recent HIV acquisition were having >1 partner in the previous 12 months (adjusted odds ratio [aOR] 1.92) and having >1 partner who thought or was told they were HIV-positive (aOR 7.25) or had unknown HIV status (aOR 2.05).

In the multivariable model, compared with those with longstanding infections, those 15–24 years of age (aOR 4.79) and 25–34 years of age (aOR 2.23) were more likely to have a recently acquired infection than were participants who were 35–49 years of age (Table 5). Participants who used a condom the last time they had sex (aOR 0.46) and had >1 partner in the previous 12 months that the participant believed or knew to be HIV-positive (aOR 0.17) were less likely to have a recent infection than a long-term infection. Region, sex, marital status, age of sexual debut, partner age differences, and community-level viremia did not significantly differ between those with recent and long-term infections.

**Table 5:** Crude and adjusted odds ratios comparing recent and long-term HIV infections in study of risk factors for HIV infections among adults in 14 countries in Africa identified by Population-Based HIV Impact Assessment surveys, 2015–2019

Category	Crude odds ratio (95% CI)	Adjusted odds ratio (95% CI)
Region		
Eastern Africa	0.82 (0.53–1.28)	1.10 (0.68–1.76)
Southeastern Africa	0.57 (0.37–0.88)	1.03 (0.61–1.73)
Southern Africa	0.57 (0.37–0.89)	1.18 (0.66–2.11)
Western Africa	Referent	Referent
Sex		
F	0.96 (0.67–1.38)	1.00 (0.61–1.65)
M	Referent	Referent
Age Group, y		
15–24	6.03 (3.81–9.53)	4.79 (2.79–8.24)
25–34	2.60 (1.69–4.01)	2.23 (1.43–3.47)
35–49	Referent	Referent
Marital Status		
Married/cohabiting	0.45 (0.30–0.68)	0.86 (0.49–1.49)
Divorced/separated/widowed	0.61 (0.35–1.05)	1.09 (0.58–2.06)
Never married	Referent	Referent
Age of Sexual Debut, y		
<18	1.49 (1.05–2.11)	1.19 (0.82–1.71)
>18	Referent	Referent
No. sexual partners in previous 12 mo		
1	Referent	Referent
>2	1.96 (1.35–2.85)	1.83 (1.17–2.86)
Condom used at last sex		
Condom used	0.51 (0.34–0.77)	0.46 (0.28–0.74)
Condom not used	Referent	Referent
HIV status of sexual partners		
>1 partner thought/told/tested HIV+	0.22 (0.10–0.49)	0.17 (0.08–0.36)
>1 partner with unknown HIV status	1.35 (0.95–1.92)	1.18 (0.80–1.76)
All partners thought/told/tested HIV–	Referent	Referent
Age difference with sexual partners		
Partners <5 years older than participant	Referent	Referent
>1 partner 5–9 years older than participant	1.09 (0.74–1.59)	1.08 (0.68–1.72)
>1 partner >10 years older than participant	0.82 (0.54–1.24)	0.84 (0.50–1.42)
Community-level viremia*		
Lowest quartile	Referent	Referent
Second quartile	0.90 (0.40–2.01)	1.01 (0.43–2.39)
Third quartile	0.72 (0.34–1.54)	0.86 (0.37–1.99)
Highest quartile	0.58 (0.28–1.19)	0.70 (0.31–1.62)

*Lowest quartile (0–25%), <39.38 viremic persons/10,000 population; second quartile (25%–50%), 39.38–122.51 viremic persons/10,000 population; third quartile (50%–75%), 122.52–297.03 viremic persons/10,000 population; highest quartile (76%–100%), >297.03 viremic persons/10,000 population.

## Discussion

We compared persons recently infected with HIV, HIV-negative persons, and persons with long-term HIV infections in 14 countries within sub-Saharan Africa by using large, nationally representative, population-based surveys. Participants living in regions of sub-Saharan Africa with higher HIV prevalence were considerably more likely to have a recent HIV infection. Similarly, participants living in communities with higher prevalence of HIV viremia had higher odds of recent HIV infection. Both associations persisted after controlling for individual risk factors. Treatment as prevention (TasP) is a primary strategy for ending the HIV epidemic and is an essential strategy within the conceptual framework of UNAIDS 95–95–95 goals (that 95% of people living with HIV/AIDS know their status, 95% of those who know their status are on treatment, and 95% of those on treatment are virally suppressed). Multiple studies have shown that persons with undetectable levels of HIV (i.e., HIV viral load <200 copies/mL blood) have essentially no risk of transmitting HIV through sex (*19**,**20*). However, population-based studies that assess the effects of TasP on HIV incidence in communities have had fewer clear outcomes; some studies found a lack of population-level effect of TasP (*21*). Persons living in areas with higher HIV prevalence or higher prevalence of viremic people living with HIV/AIDS have increased likelihood of acquiring HIV infections (*22*). This finding reinforces the potential benefits of identifying persons who are unaware of their HIV infection and enrolling them in treatment programs to achieve sustained HIV viral suppression, as well as benefits of prevention interventions, such as scale-up of HIV preexposure prophylaxis according to programmatic need.

Consistent with existing literature from sub-Saharan Africa, we found that recent HIV infection was higher in female than male participants; female participants accounted for 65% of recent infections and had ≈2 times the adjusted odds of recent HIV infection compared with male participants (*12**,**23*). The estimated proportion of new infections occurring among adolescents and young adults 15–24 years of age (31.4%) was largely consistent with UNAIDS estimates of 2 in 7 new infections occurring among that age group (*24*). We also found that >70% of new HIV infections among adolescents and young adults were acquired by female participants, in line with the 60%–80% estimates by UNAIDS (*25*). Those findings highlight the continued need for HIV prevention programs for women and girls, along with other sexual and reproductive health services. Gender disparities in HIV incidence also highlight the need to engage male patients in treatment uptake and retention efforts to further reduce infections among their female partners (*26*). Age group was not associated with risk for recent HIV infection in adjusted models, indicating the importance of HIV prevention programming across the age continuum among sexually active adolescents and adults.

Compared with participants having sex only with partners they believed or knew were negative for HIV, having a partner that the participant knew or believed was positive for HIV or a partner with unknown HIV status was associated with acquiring a recent HIV infection. The relationship was stronger for those with a partner known or believed to be positive for HIV. Still, most new infections occurred among those who had a sex partner with unknown HIV status. Very few participants with recent HIV infection had sex only with partners they thought were negative for HIV. Having sex with a partner outside of marriage, a sexual debut before turning 18 years of age, and having multiple partners were also associated with an increased risk for new HIV infection. Those findings are not surprising, because they are broadly consistent with similar analyses completed over the previous 2 decades (*12**,**13*). Although HIV incidence has decreased during that period, further declines in incidence and reduction in incidence disparities might rely on continued targeted testing to identify those persons at risk of transmitting HIV, as well as interventions that encourage disclosure of positive status, promote access to antiretroviral therapy to suppress viral load, prevent transmission, reduce the number of sexual partners, and promote safe sex and access to preexposure prophylaxis.

Certain factors could not be included in the final model because those data were collected inconsistently across countries or were applicable only to subpopulations. However, voluntary medical male circumcision has been shown to reduce the risk of HIV acquisition by 38%–66% (*27*), and our bivariate comparisons of participants with recent infections compared with HIV-negative participants are consistent with those data. Additional analyses of PHIA data from fewer countries but using methods designed to specifically determine the effect of male medical circumcision on HIV incidence have similarly found a substantial protective effect, particularly among younger men and boys 15–34 years of age (*28*).

Although using RITAs in cross-sectional surveys enables the examination of risk factors for new infections, limitations to this approach exist. PHIAs are designed to estimate national incidence rates by using RITAs and are not powered to examine any specific associations between potential risk factors and recent infections. Even though PHIAs were used in some countries with the highest HIV prevalence worldwide and sample sizes were large, very few (range 4–31) recent infections among the study populations were identified. The rarity of the outcome precluded country-specific analyses of risk factors for recent infections. In addition, cultural context, epidemic dynamics, and responses of governments to the epidemic are not homogenous across the continent. Therefore, we could not examine those nuances across or within countries. The size and scope of PHIAs prevents data being available in near real-time; thus, delays in monitoring HIV-acquisition trends using survey-based approaches exist, and risk factors for new infection might change over time. In addition, previous studies that have used a similar approach within a single country had larger sample sizes of recent infections because a larger proportion of HIV infections were classified as recent, such as 7% in Kenya (*13*) and 17% in Uganda (*12*). In contrast, only 1.6% of HIV infections in our study were classified as recent infections, likely because incidence rates have declined; the studies in Uganda (*12*) and Kenya (*13*) used data collected during 2007, whereas PHIA data used in our study were collected during 2015–2019. Furthermore, differences in RITAs might have contributed to differences in proportions of HIV infections classified as recent; for example, the Uganda study used a different assay, which might have a higher false recency rate than the LAg-avidity EIA (*29*). Moreover, PHIAs used in our study were conducted with a revised RITA that incorporated viral load (>1,000 copies/mL) and absence of antiretroviral drug metabolites.

Because this study used a pooled analysis, we were limited to factors that were collected consistently across each PHIA, reducing our ability to examine potential relationships between recent infections and other factors, such as mobility, violence, stigma, alcohol use, and sexually transmitted infection symptoms or diagnosis, which were not collected consistently across countries. Other potential risk factors, such as education, had to be regrouped into broad categories that might have limited our analysis of their relationship with recent HIV infection. Furthermore, only countries that completed a PHIA were included in the analysis; therefore, results might not be generalizable to other countries in sub-Saharan Africa. Community-level viremia was calculated at the stratum level, which often represented larger geographic or political areas such as regions or provinces. Finally, a potential for misclassification of potential risk factors in PHIAs existed because of the self-reported nature of risk factors of interest and potential for outcome misclassification by RITA.

Despite those limitations, we successfully identified key factors associated with recent infections among the adult populations of 14 countries that have high HIV burdens. Focusing prevention resources on persons who are at higher risk of acquiring a recent infection should contribute to the continued decline in HIV incidence and, ultimately, to epidemic control. Additional strategies will be needed to monitor recent infections, such as routine surveillance as part of HIV testing services for rapid case and cluster investigations (*30**–**32*) or using testing history–based methods to classify recent infections within surveys that do not require the use of a recency assay (*33*). Those data will provide actionable information for HIV programs regarding new outbreak locations and where prevention resources might be needed most (*30*).
